# Development of high-yielding white maize hybrids with better *chapatti*-making quality compared to traditionally used local landraces

**DOI:** 10.3389/fnut.2024.1330662

**Published:** 2024-02-26

**Authors:** Arushi Arora, Abhijit Kumar Das, Ramesh Kumar, Savita Sharma, Navjot Kaur, Shubhank Dixit, Yashmeet Kaur, D. C. Saxena, Sujay Rakshit

**Affiliations:** ^1^Plant Breeding and Genetics, Punjab Agricultural University, Ludhiana, India; ^2^ICAR-Indian Institute of Maize Research, PAU Campus, Ludhiana, India; ^3^Food Science and Technology, Punjab Agricultural University, Ludhiana, India; ^4^Sant Longowal Institute of Engineering and Technology, Punjab, India; ^5^ICAR-Indian Institute of Agricultural Biotechnology, Ranchi, Jharkhand, India

**Keywords:** white maize, *chapatti*, proximate composition, pasting properties, textural properties, sensory evaluation

## Abstract

**Introduction:**

The present research focuses on the chapatti making quality of high-yielding white maize hybrids compared to available low-yielding local yellow and white landraces in India.

**Materials and methods:**

In this study, the top nine superior hybrids were selected for testing the physical properties of the maize kernels, proximate composition of flours and *chapattis*, physical parameters of *chapatti*, textural properties, sensory evaluation of *chapattis* and pasting properties of maize flour.

**Results and discussion:**

The results revealed the superiority of white maize hybrids (WMH), *viz.*, WHM 1, WHM 2, and WHM 8 over the local yellow and white landraces for most of the parameters studied. In sensory analysis, though, the yellow landrace was considered superior by the panellists in terms of colour but the white maize hybrids outperformed in overall sensory analysis and were more acceptable than the yellow and white maize landraces. These high yielding white maize hybrids with good consumer acceptance may cater for the needs of rural and tribal populations in India who prefer white maize as a staple food.

## Introduction

Maize (*Zea mays* L.) is one of the important cereal crops of the world being used as food, feed, fodder and raw material for a large number of industrial products (1, 2). Based on endosperm colour maize can be broadly categorized into two widely grown types, *viz.*, yellow and white maize. White maize is the major staple food crop in parts of Africa, Central America, South America, Kenya, Malawi, Tanzania, and Zimbabwe. The majority of the maize grown in India is mostly the yellow type that is being diverted to poultry and animal feed. Maximum focus is given to yellow maize breeding programmes keeping in mind the feed industry resulting in little effort towards white maize improvement programmes which had led to less availability of high-yielding white maize hybrids. However, white maize landraces are extensively consumed by rural and tribal populations (3–5). In India, white maize is preferred by the farmers of Jammu and Kashmir, Gujarat, parts of Himachal Pradesh, Madhya Pradesh and Rajasthan. Thus, it is important to put concerted efforts into the genetic enhancement of white maize germplasm for developing high-yielding white maize hybrids and release these hybrids for commercial cultivation to cater to the needs of the people who prefer white maize as food.

White maize in India is used as a traditional cereal which requires cooking or heating before its consumption. So, it is important to focus on the quality characteristics, organoleptic traits and nutritional properties of developed products. Maize is a staple food in different parts of the country, particularly in South East-Asia, where it is consumed in the form of unleavened flatbread known as *chapatti*. These *chapatti*s are consumed as a staple diet in several parts of India (6). Thus, it is important to study the *chapatti*-making qualities of maize flour prior to its release for commercial cultivation. Fewer efforts have been made for the evaluation of such characteristics when hybrids are generally developed. However, due to traditional preferences, white maize cannot be replaced by any other crop or even by yellow maize in areas where it is devoted as a staple food. Hence, it is important to develop productive white hybrids with comparable or better organoleptic properties than the low-yielding local landraces for the betterment of the tribal and rural populations of the country. These efforts will, in turn, contribute to the improvement of the nutritional status of rural communities which are often resource-limited. With this perspective, the current study was conducted to assess the nutritional properties of superior white maize hybrids and compare these hybrids with local yellow and white landraces to assess the acceptability of the white maize hybrids.

## Materials and methods

### Plant materials

A total of 156 white maize hybrids were grown and tested at three locations, *viz.*, Ludhiana, Hyderabad and Bihar in India. Mean grain yield data for three locations for these hybrids were pooled. Out of these 156 white maize hybrids, the top nine hybrids recorded >10% superiority for grain yield over the best check. The best check recorded yield of 6206.3 kg/ha whereas all the nine hybrids recorded grain yield >6,816 kg/ha grain yield. Two local landraces (white and yellow) and the check (Bio 605) were selected for evaluation of organoleptic and nutritional properties. One desi yellow landrace (Solan-L) and one local white landrace (Mali-1) were also tested for these characterstics. Solan-L and Mali-1 are landraces that are commonly used for preparing *chapatti* in tribal pockets. A total of twelve samples were subjected in the present study (Supplementary Table S1). Out of the 12, nine of the white hybrids were generated at ICAR-IIMR, Ludhiana, Solan-L was collected from Solan (Himachal Pradesh), Mali-1 was collected from Jammu and Kashmir and Bio 605 was a hybrid from Bio seed. All the samples were sun-dried, cleaned thoroughly for any foreign contamination, dust, diseased, infested or irregular seeds analyzed for physical properties and milled to prepare maize flour (<200 μ) using the laboratory mill (Perten Instruments, Hagersten, Sweden), sieved and stored properly for further analysis.

### Physical properties of maize kernel

The maize grains were analyzed for physical quality, *viz.*, thousand kernel weight, hectoliter weight and bulk density.

### Thousand kernel weight (TKW)

The TKW was recorded by weighing a hundred grains on a micro-weighing electronic analytical balance of sartorius© and multiplied by 10. The weight of 1,000 kernels was recorded in grams (gms) as per Kaur et al. (6).

### Hectoliter weight

The weight of grains filled in a measuring cylinder of 1.0-liter capacity was recorded. The final value was noted in kilograms/ hectoliters (kg/hL) (7).

### Bulk density

The bulk density was calculated by taking 50 gms of grains in a 250 mL measuring cylinder (8). This was followed by tapping the cylinder gently ten times and the volume was noted and expressed as g/cm^3^.

### Physio-chemical analysis of maize flour and *chapatti*

#### Moisture content

The moisture content in maize flour and *chapatti* was determined using the standard air oven method. Weighed flour of finely ground grains and *chapatti* powder was subjected to the hot air oven for 3 to 4 hours at 1300 ± 10°C. The moisture content was calculated using the following formula (9):


$$\mathrm{Moisture}\;\mathrm{content}\;\left(\%\right)=\frac{\mathrm{Loss}\;\mathrm{in}\;\mathrm{moisture}\left(gm\right)}{\mathrm{Initial}\;\mathrm{weight}\;\mathrm{of}\;\mathrm{sample}\;\left(gm\right)}\times 100$$

#### Ash content

The flour and *chapatti* powder samples were taken in pre-weighed crucibles followed by charring on a hot plate. The charred samples were placed in a muffle furnace at 550°C for 6 h and were then placed in the desiccators. The weight of the final crucible is noted as the total ash content (10).


$$\begin{array}{l} \%\mathrm{Ash}\;\mathrm{content}=\left[\left(\mathrm{ashed}\;\mathrm{weight}\right)-\left(\mathrm{crucible}\;\mathrm{weight}\right)\right]\\ \;\times 100/\left(\mathrm{crucible}\;\mathrm{and}\;\mathrm{sample}\;\mathrm{weight}\right) \end{array}$$

#### Protein content

The protein content of flour and *chapatti* samples was determined using the micro-Kjeldahl method (11). Nitrogen and protein contents were calculated as per the following formula:


$$\%\mathrm{Nitrogen}=\frac{\mathrm{Titre}\;\mathrm{value}\left(\mathrm{Blank}-\mathrm{Sample}\right)\times 0.0014}{\mathrm{Weight}\;\mathrm{of}\;\mathrm{the}\;\mathrm{sample}\;\left(gm\right)}\times 100$$

$$\%\mathrm{Protein}=\%\mathrm{Nitrogen}\times \mathrm{Conversion}\;\mathrm{Factor}\;\left(6.25\right)$$

A general composite conversion factor of 6.25 was used to calculate the percent crude protein content.

#### Fat content

The fat content of the flour and *chapatti* samples was analyzed by FOSS instrument-Soxtec 2045 (Sweden). Crude fat (%) was calculated from the increase in the weight of the beakers (9):


$$\begin{array}{l} \%\mathrm{Crude}\;\mathrm{fat}=(\;\mathrm{Weight}\;\mathrm{of}\;\mathrm{extracted}\;\mathrm{fat}\;\left(gm\right)\\ -\mathrm{weight}\;\mathrm{of}\;\mathrm{empty}\;\mathrm{flask}\;\left(gm\right)\times \frac{100}{\mathrm{weight}\;\mathrm{of}\;\mathrm{sample}} \end{array}$$

#### Fiber estimation

The fiber estimation was performed using the fibertec (Foss instrument, Sweden) apparatus (12). The fiber content was calculated as per the following formula:


$$\mathrm{Fiber}\;\left(\%\right)=\frac{W3-\left(W1-S\right)-\left(W5-W4-A\right)}{W2}\times 100$$

Where,

W1 = Weight of empty capsule.
W2 = Weight of sample.
W3 = Capsule weight + extracted and dried sample weight.
W4 = Weight of empty crucible.
W5 = Weight of crucible.
S = Capsule solubility (1.001849).
A = Capsule ash.

#### Total carbohydrate content (TCC)

The TCC was calculated by using the following formula:


$$TCC=100-\left(\mathrm{moisture}+\mathrm{ash}+\mathrm{fat}+\mathrm{protein}+\mathrm{fiber}\right)$$

In the case of *chapattis*, moisture content was not taken into consideration while calculating TCC as per Sharma (13).

### Preparation of *chapattis*

The maize kernels were subjected to the milling of grains and passed through a sieve to obtain fine flour. *Chapatti* making process involved the flour being kneaded with varying proportions of warm water till smooth, cohesive, non-sticky dough was obtained and percent water absorption was noted for each hybrid to prepare dough effectively. Dough after a while was rolled into *chapattis* and baked (6).

### Percent water absorption

Lukewarm water was added to 100 gms of flour to form the smooth and non-sticky dough. The optimum amount of water added to prepare the dough was measured and noted (14).

### Physical characters of *chapatti*

The dough was divided into 40 gm of dough balls to prepare *chapattis.* The thickness and diameter of prepared *chapattis* were measured in three replicates. The diameter of the *chapattis* was measured using a scale and thickness was measured using a vernier caliper from various angles.

### Sensory evaluation

*Chapattis* were analyzed for sensory scores in terms of flavor, texture, colour, appearance and overall acceptability. *Chapattis* were presented on white papers labeled with random numbers to ten panelists. The panelists included five women and five men, of different states and ages. To test the flavor and textural feel of *chapatti*s, all panelists were blindfolded to avoid any bias. Then the blindfolds were removed to rate for colour, appearance and overall acceptability. All the panelists were instructed to rinse their mouths with water after tasting every sample. A nine-point hedonic (1–9) scale (15) where 1 stands for “extremely disliking,” 9 stands for “extremely liking” and 5 stands for “neither like nor dislike,” was used.

### Textural properties of *chapatti*

Texture Profile Analysis (TPA) of each *chapatti* strip in triplicates was evaluated using the TA/XT2 Texture analyzer (Stable Micro Systems, Surrey, England). Texture Profile Analysis (TPA) involved measuring parameters such as adhesiveness, cohesiveness, springiness, hardness, chewiness, and gumminess. The samples were cut into consistent sizes and a cylindrical aluminum (P25) probe was used to apply pressure. The instrumental conditions were as follows: Pre-test speed: 10.0 mm/min, Post-test speed: 10.0 mm/min, Trigger: 15.0 g, and Load cell: 20.0 kg (16).

### Pasting properties of maize flour

The pasting properties of maize flour samples were analyzed using a Rapid Visco Analyzer (RVA, Starch Master TM; Model: N17133; Newport Scientific Pvt. Ltd., Warriewood, Australia) following the standard AACC International Method 76-21.01. The samples were vigorously shaken up and down 10 times using the paddle inserted into the canister. The canister was placed into the instrument that had been pre-adjusted. The samples were weighed (3.00 gm) and added to the RVA canister along with 25 mL of distilled water. These samples were then heated from 50°C to 95°C at a rate of 12°C/min, held at 95°C for 5 min, and then cooled to 50°C at a rate of 12°C/min. After removing the canister from the instrument, the samples were disposed. The pasting properties including peak viscosity, trough viscosity, breakdown viscosity, final viscosity, setback viscosity, peak time, and pasting temperature were recorded (6).

### Statistical analysis

The data was collected in triplicates and represented as the mean ± standard deviation. The data were subjected to analysis of variance (ANOVA) to determine statistically significant differences (*p* < 0.05) among the samples using the F-test. Duncan Multiple Range Test (DMRT) was done for the classification of the difference between any two treatment means as significant or non-significant. For comparison of proximate characters of flour and *chapatti*, a paired *t*-test was performed. Correlation analysis was performed using Kenall’s tau correlation matrix. Data analysis was done using R software (v4.1.2: R Core Team 2021) in a Completely Randomized Design (CRD).

## Results and discussions

### Selection of white maize hybrids for food quality analysis

The top nine hybrids out of the 156 hybrids recorded >10% superiority for grain yield/ha over the best check. The CD, CV, hybrids, with their mean grain yield values and % superiority of crosses, are presented in Supplementary Table S2.

### Physical quality parameters of maize grains

The physical parameters such as TKW and bulk density are used in the assessment of the quality, yield and productivity of maize (Table 1).

**Table 1:** Physical characters of maize grains for samples used in food quality estimates.

S. No.	Sample	TKW (gm)	Bulk density (gm/ml)	Hectoliter weight (kg/hl)
1	WHM 1	263.72 ± 4.24^b^	0.764 ± 0.01^a^	74.65 ± 4.34^a^
2	WHM 2	293.57 ± 4.94^a^	0.722 ± 0.01^a^	73.10 ± 3.59^a^
3	WHM 3	247.39 ± 2.12^de^	0.722 ± 0.01^a^	76.75 ± 3.48^a^
4	WHM 4	253.58 ± 4.24^cd^	0.746 ± 0.01^a^	75.75 ± 3.46^a^
5	WHM 5	223.39 ± 4.95^g^	0.781 ± 0.02^a^	76.90 ± 3.04^a^
6	WHM 6	237.33 ± 2.83^f^	0.758 ± 0.01^a^	74.75 ± 2.76^a^
7	WHM 7	243.91 ± 4.24^ef^	0.736 ± 0.03^a^	75.85 ± 2.62^a^
8	WHM 8	256.54 ± 2.82^bcd^	0.762 ± 0.04^a^	73.35 ± 0.56^a^
9	WHM 9	224.65 ± 5.65^g^	0.764 ± 0.03^a^	79.25 ± 2.06^a^
10	Solan-L (Yellow landrace)	260.69 ± 2.12^bc^	0.782 ± 0.02^a^	77.85 ± 2.93^a^
11	Mali-1 (White landrace)	254.85 ± 5.65^bcd^	0.756 ± 0.03^a^	76.25 ± 3.44^a^
12	Bio 605 (Check)	250.93 ± 2.12^de^	0.731 ± 0.04^a^	73.90 ± 2.78^a^
Range		223.39–293.57	0.722–0.782	73.10–79.25
Mean		250.88	0.752	75.70

TKW, thousand kernel weight, values are presented as mean ± standard deviation, in the same column, means with different alphabets in superscript indicate significant differences (*p* ≤ 0.001), Solan-L = Yellow landrace, Mali-1 = White landrace, Bio 605 = check variety.

The TKW varied between 223.39–293.57 gm with an average mean of 250.88 gm. WHM 2 (293.57 gm) showed significantly higher TKW than the local landraces. The maize hybrids with TKW of more than 290 gm are useful for industrial purposes as they are known to provide higher yields in the manufacturing of several products (6). TKW is positively correlated with kernel weight increment in achieving maximum grain filling rates (17). The bulk density varied between 0.722–0.782 gm/ml with an average of 0.752 gm/ml. Sandhu et al. (18) reported quite similar results with the bulk density range of 0.645–0.774 gm/ml. The hybrid WHM 5 (0.781 gm/ml) had a comparable bulk density to the yellow landrace, Solan-L (0.782 gm/ml). WHM 1 (0.764 gm/ml) and WHM 6 (0.758 gm/ml) had higher but non-significant bulk density than the white landrace, Mali-1 (0.756 gm/ml). The range of hectoliter weight in the study ranges between 73.1–79.25 kg/hL, the average being 75.70 kg/hL. For hectoliter weight, WHM 9 (79.25 kg/ hL) performed non-significantly better than yellow landrace (77.85 kg/hL), whereas, WHM 3 (76.75 kg/hL) and WHM 5 (76.90 kg/hL) had higher hectoliter weight than white landrace (76.25 kg/hL). High hectoliter weight is generally associated with a higher density of maize kernels and thus, a higher market has a value of the grains (19). Kara (20) reported that seed size also has a significant effect (*p* < 0.05) on hectoliter weight and the highest hectoliter weight (78.7 kg) were obtained from large seeds.

### Proximate compositional analysis of flour

The white maize hybrids varied significantly in terms of proximate compositional parameters (Table 2). The moisture content varied between 4.03–9.50% with an average of 6.97%, which is quite lesser than the ideal average value of moisture content (10.23%) in maize (21). In terms of moisture content, yellow landrace (4.03%) recorded the least moisture content. However, six white maize hybrids had lower moisture contents as compared to the white landrace (7.20%). The flour with lower moisture content has a longer shelf life because it is less likely to spoil due to the growth of microorganisms or other biochemical reactions (22). In the study, the moisture content of all the white maize hybrids was less than 10% indicating, they are fit for long-term storage. Ash content specifically relates to the mineral composition found within a food substance and serves as an indicator of the overall mineral constituents present within food products (23). The ash content varied between 1.30–2.27% with an average of 1.67% Enyisi et al. (24) also reported a similar range of ash content in maize flour, 1.10–2.95%. WHM 7 (2.27%) and WHM 8 (2%), had significantly the highest ash content as compared to both yellow landrace (1.63%) and white local landrace (1.70%). Hybrids with high ash contents are positively correlated to the inorganic nutrient contents in foods. Thus, consumption of these hybrids will provide higher nutritive values (25).

**Table 2:** Proximate compositional analysis of flour.

S. No.	Sample	Moisture (%)	Ash (%)	Fat (%)	Protein (%)	Fiber (%)	TCC (%)
1	WHM 1	5.87 ± 0.15^d d^	1.30 ± 0.01^h^	4.83 ± 0.30^bc^	9.40 ± 0.40^ab^	1.83 ± 0.20^ab^	76.77 ± 0.50^bc^
2	WHM 2	8.73 ± 0.21^b^	1.40 ± 0.03^gh^	4.47 ± 0.41^cde^	9.31 ± 0.36^b^	1.30 ± 0.26^bc^	74.79 ± 0.53^efg^
3	WHM 3	9.43 ± 0.15^a^	1.30 ± 0.20^h^	4.40 ± 0.26^cde^	9.10 ± 0.10^bc^	1.90 ± 0.30^a^	73.87 ± 0.64^fg^
4	WHM 4	7.73 ± 0.38^c^	1.80 ± 0.20^bcd^	4.90 ± 0.10^bc^	8.73 ± 0.25^cd^	1.70 ± 0.17^ab^	75.13 ± 0.60^de^
5	WHM 5	6.20 ± 0.20^d^	1.71 ± 0.05^cde^	4.63 ± 0.23^bcd^	9.87 ± 0.32^a^	1.10 ± 0.10^c^	76.50 ± 0.64^bcd^
6	WHM 6	6.37 ± 0.40 ^d^	1.57 ± 0.05^efg^	4.17 ± 0.12^de^	9.34 ± 0.20^b^	1.03 ± 0.20^c^	77.53 ± 0.26^ab^
7	WHM 7	6.23 ± 0.25 ^d^	2.27 ± 0.15^a^	5.10 ± 0.26^b^	8.69 ± 0.25^cd^	2.27 ± 0.30^a^	75.45 ± 0.63^cde^
8	WHM 8	6.07 ± 0.20 ^d^	2.00 ± 0.10^b^	6.00 ± 0.20^a^	9.13 ± 0.15^bc^	1.77 ± 0.26^ab^	75.04 ± 0.63^ef^
9	WHM 9	6.23 ± 0.41 ^d^	1.50 ± 0.10^fgh^	4.13 ± 0.25^de^	8.53 ± 0.35^d^	1.93 ± 0.15^a^	77.67 ± 1.00^ab^
10	Solan-L (Yellow Landrace)	4.03 ± 0.45^e^	1.63 ± 0.05 ^def^	4.77 ± 0.32^bc^	9.33 ± 0.15^b^	1.97 ± 0.32^a^	78.27 ± 0.25^a^
11	Mali-1 (White Landrace)	7.20 ± 0.56^c^	1.70 ± 0.10^def^	4.03 ± 0.21^e^	9.23 ± 0.20^bc^	1.90 ± 0.26^a^	75.94 ± 1.00^cde^
12	Bio 605 (Check)	9.50 ± 0.44^a^	1.90 ± 0.05^bc^	4.20 ± 0.26^de^	9.03 ± 0.28^bcd^	1.90 ± 0.21^a^	73.47 ± 1.05^g^
Range		4.03–9.50	1.30–2.27	4.03–6.00	8.53–9.87	1.03–2.27	73.47–78.27
Mean		6.97	1.67	4.64	9.14	1.72	75.87

TCC, total carbohydrate content, values are presented as mean ± standard deviation, in the same column, means with different alphabets in superscript indicate significant differences (*p* ≤ 0.001), Solan-L = Yellow landrace, Mali-1 = White landrace, Bio 605 = check variety.

The fat content ranged between 4.03–6.00% and the average being 4.64% which is more than the average fat content in normal maize, 4.57% (21). WHM 8 (6.00%) had significantly higher fat content than yellow landrace (4.77%). However, all white maize hybrids had higher (non-significant) fat content than white landrace (4.03%). Maize flour that contains a higher fat content can enhance the energy content of the flour and thereby, serve as a valuable source of essential fatty acids (26).

Protein is a vital component of food and people who follow higher-protein diets tend to lose more weight, body fat and preserve more lean muscle mass than those who follow lower-protein diets (27). The protein content varied between 8.53–9.87% with an average of 9.14% protein content. WHM 5 (9.87%) had significantly higher protein content than both the yellow landrace (9.33%) and white (9.23%) landraces. Siyuan et al. (28) also studied the nutritional traits of white maize hybrids and found the average protein content as 9.42%.

Fiber also affects digestive health, aids in weight management, management of cardiovascular health and the prevention of chronic diseases (29). Fiber content of samples ranged between 1.03–2.27% with an average fiber content of 1.72%. WHM 7 (2.27%) had a higher (non-significant) value than the yellow (1.97%) and white landrace (1.90%). Similarly, Qamar et al. (30) reported a range of 0.95–2.01% fiber content in white maize flour.

Foods rich in carbohydrates are essential for a well-balanced diet as they supply glucose to the body (31). The TCC ranged between, 73.47–78.27% with an average of 75.87%. Qamar et al. (30) reported similar results with the TCC of white maize flour in the range of 65.38–78.74%. No white maize hybrid performed significantly higher than yellow landrace (78.27%) in terms of TCC. Although, WHM 6 (77.53%) and WHM 9 (77.67%) had significantly higher TCC than the white landrace (75.94%).

### Characteristics physical parameters of *chapatti*

Physical parameters like water absorption capacity of flour, while preparing *chapattis*, their thickness, diameter and baking time were performed (Table 3). The baking time of the *chapattis* ranged between 1.14–2.50 min with an average of 1.64 min. The baking time of *chapattis* made from whole wheat (16) also ranged between 2.00–3.00 min, slightly higher than the present study. The highest baking time was required by the check, Bio 605 (2.50 min) followed by yellow landrace (2.32 min), white land race (2.17 min), and WHM 6 (2.05 min). Whereas, WHM 2 had the lowest baking time (1.14 min). The higher overall acceptability of white maize hybrids was correlated to a lesser baking time. The rationale behind this is that stronger dough often requires longer baking times, which may result in tougher *chapattis* with lower acceptability (32).

**Table 3:** Physical parameters of *chapattis.*

S. No.	Sample name	Overall acceptability	Baking time (minutes)	Water absorption capacity (ml/100 g)	Thickness (mm)	Diameter (cm)
1	WHM 1	7.21 ± 0.28^a^	1.45 ± 0.06^de^	139.33 ± 1.15^bcd^	3.70 ± 0.42	10.57 ± 0.06^fg^
2	WHM 2	6.22 ± 0.12^c^	1.14 ± 0.05^g^	116.33 ± 2.51^g^	3.17 ± 0.31	11.60 ± 0.40^bcd^
3	WHM 3	5.2 ± 0.17^g^	1.22 ± 0.04^fg^	127.33 ± 6.80^ef^	3.75 ± 0.31	11.17 ± 0.31^cdef^
4	WHM 4	5.78 ± 0.91^ef^	1.31 ± 0.19^ef^	144.33 ± 4.04^b^	3.76 ± 0.40	10.67 ± 0.21^efg^
5	WHM 5	5.00 ± 0.29^g^	1.52 ± 0.04^d^	139.00 ± 6.00^bcd^	2.36 ± 0.28	10.20 ± 0.26^g^
6	WHM 6	5.61 ± 0.25^f^	2.05 ± 0.13^c^	158.00 ± 2.65^a^	3.07 ± 0.37	10.57 ± 0.21^fg^
7	WHM 7	5.59 ± 0.33^f^	1.21 ± 0.02^fg^	125.33 ± 4.72^f^	2.96 ± 0.13	11.13 ± 0.15^def^
8	WHM 8	6.57 ± 0.21^b^	1.24 ± 0.02^fg^	132.67 ± 2.51^def^	1.58 ± 0.23	11.67 ± 0.51^bcd^
9	WHM 9	6.01 ± 0.21^cde^	1.50 ± 0.04^d^	133.33 ± 2.88^def^	2.98 ± 0.13	11.40 ± 0.10^cde^
10	Solan-L (Yellow Landrace)	6.03 ± 0.31^cd^	2.32 ± 0.10^b^	142.67 ± 2.51^bc^	2.52 ± 0.27	12.30 ± 0.20^ab^
11	Mali-1 (White Landrace)	6.21 ± 0.28^c^	2.17 ± 0.05^bc^	115.33 ± 8.38^g^	2.80 ± 0.26	11.97 ± 0.87^abc^
12	Bio 605 (Check)	5.94 ± 0.21^de^	2.50 ± 0.06^a^	134.67 ± 4.50^cde^	1.84 ± 0.27	12.73 ± 0.75^a^
Range		5.00–7.21	1.14–2.50	115.33–158.00	1.58–3.76	10.20–12.73
Mean		5.95	1.64	134.03	2.80	11.33

Values are presented as mean ± standard deviation, in the same column, means with different alphabets in superscript indicate significant differences (*p* ≤ 0.001), Solan-L = Yellow landrace, Mali-1 = White landrace, Bio 605 = check variety.

The amount of water that dough can absorb is a key factor that affects its handling and sheeting properties (33). The average water absorption capacity ranged from, 115.33–158.00 mL/100 gm with an average of 134.03 mL/100 gm. However, water absorption capacity was much lower in the case of *chapattis* prepared from composite flours with values between 70 and 90 mL/100 gm as reported by Tangariya et al. (34). The highest water absorption by flour during dough formation was reported by, WHM 6 (158 mL/100 gm). Thus, WMH 6 exhibits enhanced water retention capabilities during the baking process, resulting in a desirable soft texture in the final product. This further, suggests that this particular genotype has absorbed a significant amount of water, which is advantageous for the baking of *chapattis* (35). The white landrace (115.33 mL/100gm) had the lowest water absorption followed by WHM 2 (116.33 mL/100gm). However, WHM 1 having the highest acceptability rate (7.21) showed moderate water absorption capacity (139 mL/ 100 gm). An appropriate amount of water helps the dough to come together into a smooth, workable mass during kneading. This makes it easy to shape the dough into *chapattis* but too much water will make the dough sticky and difficult to handle (36, 37).

The thickness of *chapattis* ranged between, 1.58–3.76 mm with an average of 2.80 mm. Yadav et al. (16) obtained an average thickness of *chapattis* between 2.5–4.5 mm, which is quite higher than the *chapattis* prepared in the present study from white maize hybrids samples. WHM 4 (3.76 mm) rolled into the thickest *chapatti.* Its overall acceptability was lower than average. WHM 8 (1.58 mm) had the thinnest *chapatti* without breaking and the overall acceptability of the hybrid was more than the average value. Moreover, the white maize hybrids with the highest overall acceptability, WHM 1 (3.70 mm) and WHM 2 (3.17 mm) had thicker *chapattis*. The results suggest that panelists do prefer slightly thick *chapattis* prepared from maize flour. The *chapattis* with a thicker consistency were also softer and comfortably chewable, while those with a thinner consistency tended to turn crispier and delicate. The diameter of these *chapatti*s ranged from 10.20 cm – 12.73 cm with an average of 11.33 cm. The hybrid with the highest diameter of *chapatti* was WHM 8 (11.67 cm), without showing cracks. Its overall acceptability was also more than the average value. However, the highest diameter of *chapattis* was recorded by check, Bio 605 (12.73 cm) followed by yellow landrace (12.30 cm).

### Proximate compositional analysis of *chapatti*

The white maize hybrids varied significantly in terms of proximate compositional parameters for *chapattis* (Table 4). The moisture content increases during the preparation of *chapattis* and it is directly proportional to the softness of *chapattis* (6). The moisture content for *chapatti*s ranged between 29.90–44.47% with an average of 41.04%. WHM 8 (44.47%) followed by WHM 9 (44.23%) had significantly higher moisture content than yellow landrace (39.40%). Whereas, only WHM 8 (44.47%), had significantly higher moisture content than white landrace (40.00%).

**Table 4:** Proximate composition analysis of *chapatti.*

S. No.	Sample	Moisture	Ash	Fat	Protein	Fiber	TCC
1	WHM1	43.33 ± 1.15^abc^	1.33 ± 0.11^cd^	3.33 ± 0.15^d^	9.47 ± 0.21^b^	1.40 ± 0.17^de^	84.47 ± 0.14^ab^
2	WHM 2	40.73 ± 0.70^abc^	1.90 ± 0.15^a^	3.60 ± 0.30^cd^	9.43 ± 0.21^b^	1.53 ± 0.21^cd^	83.53 ± 0.45^c^
3	WHM 3	29.90 ± 5.20^d^	1.70 ± 0.20^abc^	3.73 ± 0.21^bcd^	9.33 ± 0.15^b^	1.93 ± 0.0.15^ab^	83.33 ± 0.61^c^
4	WHM 4	40.50 ± 1.90^abc^	1.50 ± 0.15^bcd^	3.43 ± 0.31^d^	9.53 ± 0.25^b^	1.53 ± 0.06^cd^	84.00 ± 0.51^bc^
5	WHM 5	42.07 ± 2.70^abc^	1.93 ± 0.15^a^	4.13 ± 0.11^ab^	10.15 ± 0.18^a^	1.07 ± 0.23^e^	82.72 ± 0.53^d^
6	WHM 6	43.83 ± 1.60^ab^	1.73 ± 0.12^ab^	3.93 ± 0.20^bc^	9.50 ± 0.17^b^	1.10 ± 0.00^e^	83.73 ± 0.15^c^
7	WHM 7	42.20 ± 1.90^abc^	1.17 ± 0.15^d^	4.43 ± 0.11^a^	8.73 ± 0.21^de^	1.80 ± 0.10^ab^	83.87 ± 0.35^bc^
8	WHM 8	44.47 ± 0.58^a^	1.33 ± 0.15^cd^	3.67 ± 0.23^cd^	9.23 ± 0.20^bc^	1.67 ± 0.11^bc^	84.10 ± 0.59^bc^
9	WHM 9	44.23 ± 1.46^ab^	1.43 ± 0.20^cd^	3.43 ± 0.30^d^	8.37 ± 0.31^e^	1.73 ± 0.11^abc^	85.03 ± 0.23^a^
10	Solan-L (Yellow Landrace)	39.40 ± 1.55^c^	1.20 ± 0.15^d^	3.37 ± 0.23^d^	8.87 ± 0.32^cd^	1.93 ± 0.06^a^	84.63 ± 0.40^ab^
11	Mali-1 (White Landrace)	40.00 ± 1.27^bc^	1.43 ± 0.20^cd^	3.70 ± 0.20^cd^	9.30 ± 0.26^bc^	1.53 ± 0.05^cd^	84.03 ± 0.32^bc^
12	Bio 605 (Check)	41.80 ± 1.30^abc^	1.43 ± 0.11^bcd^	3.77 ± 0.15^bcd^	9.30 ± 0.17^bc^	1.73 ± 0.06^abc^	83.77 ± 0.15^bc^
Range		29.90–44.47	1.17–1.93	3.33–4.43	8.37–10.15	1.07–1.93	82.72–84.63
Mean		41.04	1.51	3.71	9.27	1.58	83.93

TCC, total carbohydrate content, values are presented as mean ± standard deviation, in the same column, means with different alphabets in superscript indicate significant differences (*p* ≤ 0.001) Solan-L = Yellow landrace, Mali-1 = White landrace, Bio 605 = check variety.

The ash content in *chapattis* varied between 1.17–1.93% with an average of 1.51%. WHM 5 (1.93%) followed by WHM 2 (1.90%) and WHM 6 (1.73%), had significantly higher ash content in *chapattis* than that of yellow landrace (1.20%) and white landrace (1.43%).

The fat content in *chapattis* ranged between 3.33–4.43% and the average being 3.71%. WHM 7 (4.43%) followed by WHM 5 (4.13%), had significantly higher fat content in their *chapattis* than the yellow landrace (3.37%) and white landrace (3.70%). Research also suggests that the perception of fat in the mouth is intricately connected to the processing of taste signals in the brain (38, 39).

The range of protein in *chapattis* varied between the ranges of 8.37–10.15% with an average of 9.27% protein content. *Chapattis* prepared from WHM 5 (10.15) had significantly highest protein content followed by WHM 4 (9.53%), WHM 6 (9.50%) and WHM 1 (9.47%), than yellow landrace (8.87%). The fiber content in *chapatti*s ranged between 1.07–1.93% with an average fiber content of 1.58%. WHM 3 (1.93%) had similar fiber content to yellow landrace (1.93%). WHM 3 (1.93%) had significantly highest fiber content followed by WHM 7 (1.80%) than the white landrace (1.53%).

**Figure 1:**
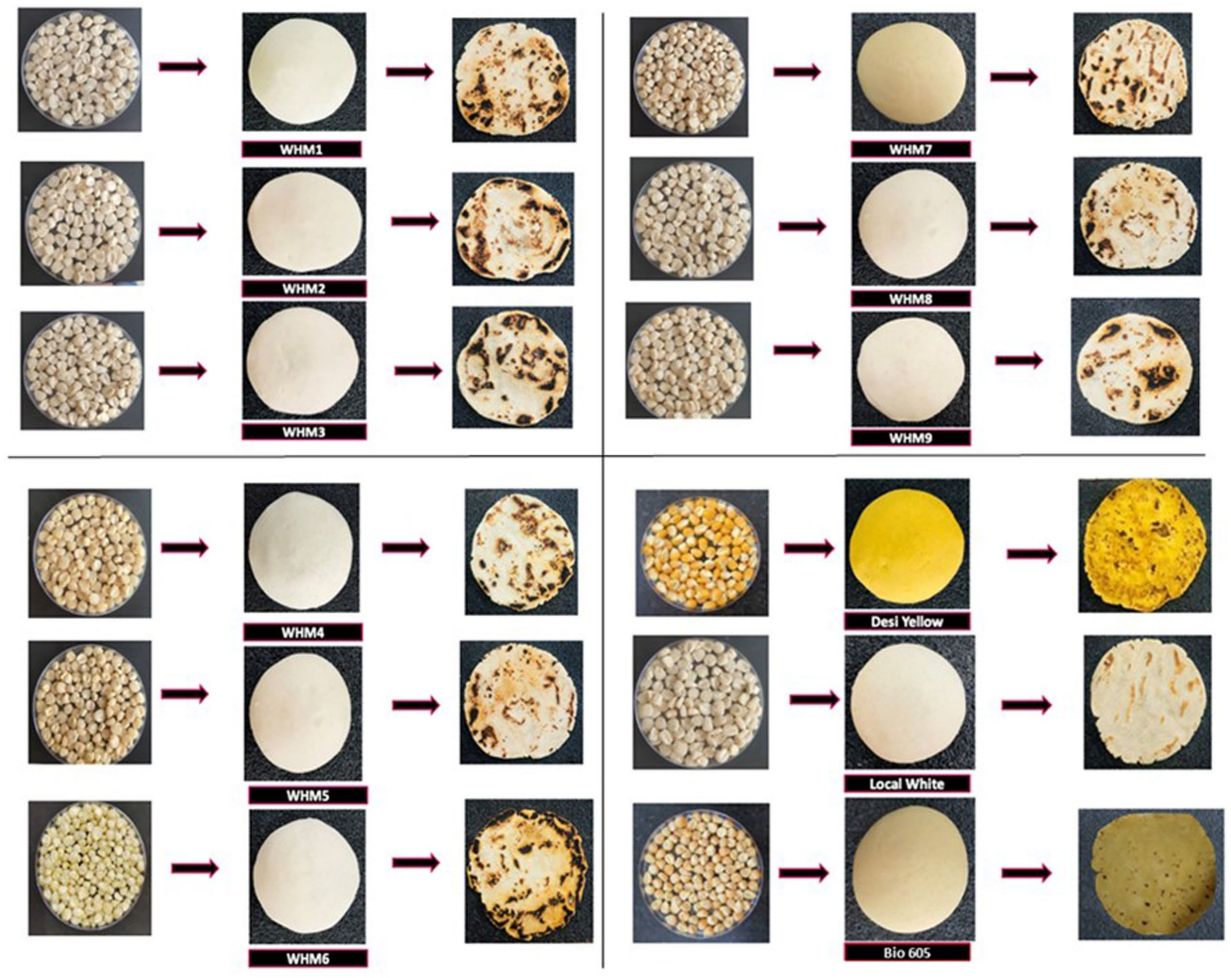
The picture shows grains, doughs and *chapattis* in the process of preparing *chapattis*.

The TCC in the *chapatti*s ranged between, 82.72–84.63% with an average of 83.93%. Yadav et al. (13) also reported TCC in *chapattis* as high as 83.37% similar to the average TCC (83.93%) in the present study. WHM 9 (85.03%) recorded a non-significantly higher TCC than the yellow landrace (84.63%) and significantly higher than the white landrace (84.03%). The pictures of grains, dough and *chapattis* are presented in Figure 1.

### Comparison of proximate composition between flour and *chapatti*

The comparison of flour and *chapatti* was performed using a *t*-test (Supplementary Table S3). During the process of preparing *chapattis*, there is a significant increase in the moisture content which is desirable for their softness. Whereas, fat and fiber content significantly decreased during the *chapatti*-making process mainly due to the biochemical degradations of these components during the preparation of *chapattis* (13). It is well-reported that this process of roasting decreases the fat content in *chapattis* prepared from maize flour (40). Moreover, the fat content in maize flour can be affected by the breakdown of the bonds in the fat molecule, within the maize matrix due to heat, leading to the effective release and mobilization of oil reserves in the maize grain following the process of roasting (41). However, there were no significant differences in protein and ash content between the flour and *chapatti* in the present study. However, there was a significant increase in the TCC in *chapattis* compared to the flours of respective samples. In a similar study by Kumar et al. (42), it was observed that an increase in carbohydrate content after roasting could potentially be attributed to the reduction in fat, protein, ash and moisture levels that occur during the roasting process.

### Sensory evaluation of *chapatti-*making qualities

A total of ten panelists evaluated the *chapatti* samples. These panelists were from different backgrounds. The testing of attributes such as flavor and mouth feel was performed by blindfolding the panelists to avoid any biases. The white maize hybrids performed significantly better than the landraces (yellow and white) and were considered superior in terms of the particular attributes. For the sensory attribute, the appearance ranged from 5.40–7.50 on a scale of 1 to 9 with an average of 6.59. Though WHM 1 (7.50) performed significantly better in terms of their appearance over the white landrace, none of the white hybrids showed significant superiority over the yellow landrace. Kaur et al. (43) also observed a similar range of appearance, 4.60–7.60. Whereas, colour ranged from 5.70–7.30 with an average of 6.44. In terms of colour of *chapattis*, no white maize hybrid performed better than yellow landrace (7.30). Though, WHM 7 (6.90) followed by, WHM 2 (6.70), WHM 1 (6.40) and WHM 4 (6.40), performed better than the white landrace (6.10) in terms of their colour but the difference was not significant. Tangariya et al. (34) obtained an average value for colour of 7.21 while performing sensory analysis of *chapattis* prepared from composite flours. Flavor values ranged between, 5.40–7.70 with an average of 6.10. Interestingly, WHM 1 (7.70), performed significantly better than the yellow (5.90) and white landrace (5.80) in terms of this attribute of flavor. The mouth feels attribute ranged between, 5.50–7.60 on a scale of 1 to 9 with an average of 6.15. WHM 1 (7.60) was significantly better as compared to the yellow (6.00) and white landrace (6.40) in terms of mouth feel. Though WHM 8 (6.90) was numerically superior over the yellow (6.0) and white landrace (6.4) the difference was non-significant.

The overall acceptability ranged between 5.00–7.21 with an average of 5.95. Considering the overall acceptability, WHM 1 (7.21) and WHM 8 (6.57) were significantly better than both the yellow (6.03) and white landrace (6.21). Kaur et al. (6) reported quite similar mean values of colour, taste/flavor and overall acceptability as 5.80, 5.90 and 5.70, respectively during the sensory analysis of maize flatbreads. Overall, WHM 1 performed significantly better as compared to the landraces. WHM 1 also had the highest acceptability in terms of *chapatti* as ranked by the panelists coupled with its highest grain yield. The yellow landrace was rated highest in terms of colour but did not outperform in terms of overall acceptability (Table 5). Thus, the blindfolding technique removed biases for other sensory parameters for hybrids and landraces.

**Table 5:** Sensory attributes for hybrids, landraces and check variety for *chapatti*-making quality.

S. No.	Sample	Appearance	Colour	Flavor	Mouth feel	Overall acceptability
1	WHM 1	7.50 ± 0.71^a^	6.40 ± 1.42^abc^	7.70 ± 0.82^a^	7.60 ± 0.51^a^	7.21 ± 0.28^a^
2	WHM 2	7.00 ± 0.94^ab^	6.70 ± 1.41^abc^	6.00 ± 1.49^b^	5.70 ± 1.06^c^	6.22 ± 0.12^c^
3	WHM 3	6.10 ± 1.37^bc^	6.20 ± 0.92^abc^	6.0 ± 0.67 ^b^	6.10 ± 1.19^bc^	5.2 ± 0.17^g^
4	WHM 4	6.30 ± 1.15^bc^	6.40 ± 1.07^abc^	5.5 ± 0.85 ^b^	5.90 ± 1.29^bc^	5.78 ± 0.91^ef^
5	WHM 5	6.50 ± 0.85^ab^	6.20 ± 1.31^abc^	5.9 ± 1.19 ^b^	5.70 ± 1.34^c^	5.00 ± 0.29^g^
6	WHM 6	5.40 ± 0.70^c^	5.70 ± 0.95^c^	5.9 ± 1.37 ^b^	6.00 ± 1.05^bc^	5.61 ± 0.25^f^
7	WHM 7	7.00 ± 0.67^ab^	6.90 ± 0.74^abc^	5.4 ± 1.17 ^b^	5.50 ± 1.18^c^	5.59 ± 0.33^f^
8	WHM 8	7.00 ± 0.81^ab^	6.00 ± 1.25^bc^	6.2 ± 1.13 ^b^	6.90 ± 0.73^ab^	6.57 ± 0.21^b^
9	WHM 9	6.40 ± 0.97^bc^	6.30 ± 1.25^abc^	6.5 ± 0.71 ^b^	6.40 ± 0.97^bc^	6.01 ± 0.21^cde^
10	Solan-L (Yellow landrace)	6.90 ± 1.19^ab^	7.30 ± 0.82^a^	5.9 ± 1.10 ^b^	6.00 ± 1.05^bc^	6.03 ± 0.31^cd^
11	Mali-1 (White landrace)	6.30 ± 1.06^bc^	6.10 ± 0.87^abc^	5.8 ± 0.79 ^b^	6.40 ± 0.70^bc^	6.21 ± 0.28^c^
12	Bio 605 (Check)	6.70 ± 0.48^ab^	7.10 ± 0.74^ab^	5.4 ± 1.07 ^b^	5.60 ± 1.17^c^	5.94 ± 0.21^de^
Range		5.40–7.50	5.70–7.30	5.40–7.70	5.50–7.60	5.00–7.21
Mean		6.59	6.44	6.10	6.15	5.95

Values are presented as mean ± standard deviation, in the same column, means with different alphabets in superscript indicate significant differences (*p* ≤ 0.001), Solan-L = Yellow landrace, Mali-1 = White landrace, Bio 605 = check variety.

### Texture profile analysis of *chapattis*

The textural properties analyzed by the texture analyzer were correlated with the overall acceptability (Table 6). The hardness ranged between 5.15–13.41 N with a mean of 8.44 N. WHM 9 showed the lowest hardness of 5.15 N whereas, WHM 3 had the highest value of hardness, 13.91 N. WHM 3 with the highest hardness had lower acceptability and WHM 9 with the lowest hardness had a higher acceptability rate. WHM 1 with the highest acceptability rate had a moderate level of hardness and the local yellow and white landrace had lower hardness. This suggests that the consumers prefer *chapattis* of maize with a lower to moderate level of hardness. However, the hardness in the texture of *chapattis* is attributed to increased viscosity due to the absence of gluten in maize. However, maize *chapattis* are good for consumers having gluten intolerance or celiac disease (44). The springiness ranged between 0.48–0.74 cm/mm with a mean of 0.64 cm/mm. WHM 9 had the lowest springiness, 0.48 cm/mm. Yellow landrace had the highest springiness of 0.74 cm/mm followed by WHM 4 and WHM 7, both with similar values of 0.72 cm/mm. However, WHM 1 and WHM 2 with the highest overall acceptability have moderate values of springiness. The cohesiveness ranges between the ratio of 0.19–0.31 with the mean ratio of 0.26. The lowest cohesiveness was found in WHM 3 with a ratio of 0.19, whereas WHM 9 has the highest value of cohesiveness, 0.31. The overall acceptability of WHM 9 is more than the mean acceptability rate. However, WHM 1 and WHM 2 with the highest overall acceptability have lower value of cohesiveness.

**Table 6:** Textural properties of *chapattis.*

S. No.	Sample	OA	Hardness (*N*)	Springiness (cm/mm)	Cohesiveness (ratio)	Gumminess (*N*)	Chewiness (*N*)	Resilience
1	WMH 1	7.21 ± 0.28^a^	9.50 ± 0.94^c^	0.63 ± 0.07^cdef^	0.22 ± 0.04^cd^	19.30 ± 0.61^cd^	10.80 ± 0.96^c^	0.09 ± 0.00^a^
2	WMH 2	6.22 ± 0.12^c^	7.20 ± 0.50^de^	0.68 ± 0.03^abc^	0.23 ± 0.02^cd^	19.67 ± 0.58^cd^	11.43 ± 0.60 ^c^	0.08 ± 0.01^a^
3	WMH 3	5.2 ± 0.17^g^	13.41 ± 1.29^a^	0.59 ± 0.01^def^	0.19 ± 0.01^d^	23.10 ± 2.10^b^	19.63 ± 0.64^a^	0.08 ± 0.01^a^
4	WMH 4	5.78 ± 0.91^ef^	10.32 ± 0.42^c^	0.72 ± 0.03^a^	0.28 ± 0.17^ab^	14.90 ± 0.80^e^	11.43 ± 0.66 ^c^	0.10 ± 0.01^a^
5	WMH 5	5.00 ± 0.29^g^	12.22 ± 0.44^b^	0.59 ± 0.06^ef^	0.28 ± 0.02^ab^	23.87 ± 1.59^b^	15.95 ± 1.29^b^	0.09 ± 0.00^a^
6	WMH 6	5.61 ± 0.25^f^	7.27 ± 0.46^de^	0.71 ± 0.04^ab^	0.26 ± 0.02^bc^	20.87 ± 1.62^bc^	12.80 ± 1.93 ^c^	0.11 ± 0.01^a^
7	WMH 7	5.59 ± 0.33^f^	10.40 ± 0.61^c^	0.72 ± 0.03^a^	0.29 ± 0.01^ab^	31.10 ± 1.09^a^	20.00 ± 1.04^a^	0.11 ± 0.01^a^
8	WMH 8	6.57 ± 0.21^b^	7.10 ± 0.40^de^	0.67 ± 0.02^abcd^	0.23 ± 0.02^cd^	17.67 ± 1.48^cde^	11.67 ± 0.76 ^c^	0.08 ± 0.01^a^
9	WMH 9	6.01 ± 0.21^cde^	5.15 ± 0.56^f^	0.48 ± 0.02^g^	0.31 ± 0.03^a^	16.83 ± 2.60^de^	21.80 ± 1.61^a^	0.09 ± 0.00^a^
10	Solan-L	6.03 ± 0.31^cd^	5.96 ± 0.85^ef^	0.74 ± 0.04^a^	0.25 ± 0.03^bc^	19.67 ± 1.52^cd^	11.30 ± 1.35 ^c^	0.08 ± 0.01^a^
11	Mali-1 (White Landrace)	6.21 ± 0.28^c^	5.95 ± 0.53^f^	0.55 ± 0.06^fg^	0.29 ± 0.02^ab^	23.67 ± 2.37^b^	13.10 ± 1.51 ^c^	0.07 ± 0.01^a^
12	Bio 605	5.94 ± 0.21^de^	7.88 ± 0.90^d^	0.64 ± 0.05^bcde^	0.29 ± 0.02^ab^	23.53 ± 2.10^b^	13.47 ± 2.48 ^c^	0.04 ± 0.00^a^
Range		5.00–7.21	5.15–13.41	0.48–0.74	0.19–0.31	14.9–31.10	10.80–21.80	0.04–0.11
Mean		5.95	8.44	0.64	0.26	21.35	14.78	0.09

OA, overall acceptability; values are presented as mean ± standard deviation, *n* the same column, means with different alphabets in superscript indicate significant differences (*p* ≤ 0.001), Solan-L = Yellow landrace, Mali-1 = White landrace, Bio 605 = check variety.

WHM 4 had the lowest gumminess, 14.90 N, whereas, WHM 7 possessed the highest gumminess, 31.10 N. The gumminess values ranges between 14.90N–31.10N. The acceptability of both these white maize hybrids falls in the average category. The gumminess of white maize hybrids with the highest overall acceptability, WHM 1 and WHM 2 had a little lower value than the average gumminess. This indicates that consumers prefer a little lesser gumminess in *chapatti*s of white maize hybrids. In the case of *chapatti*, gumminess is the extent of resistance offered by the *chapatti* during biting and chewing. It depends on the cohesiveness and hardness of the dough (45). WHM 1 had the lowest chewiness, 10.80 N and WHM 9 had the highest chewiness, 21.80 N. The chewiness varied between 10.80–21.80 N and the average being 14.78 N. Interestingly, WHM 1 with the lowest chewiness, possesses the highest acceptability by the panelists. This links the higher acceptability of white maize *chapattis* with a low chewiness for acceptance by consumers. El-Sohaimy et al. (46) while evaluating the sensory characteristics of flat bread supplemented with the flour of quinoa, reported that increasing the gumminess and chewiness in the flatbread causes a decrease in the organoleptic scores when compared to normal wheat bread.

WHM 6 and WHM 7 had the highest resilience, 0.11, whereas the lowest level of resilience was found in the white landrace (0.07) and the check, Bio 605 (0.04). WHM 2, WHM 3 and WHM 8 had the lowest resilience, 0.08. Although, there was no direct correlation between resilience and overall acceptability. However, the white maize hybrids with a high resilence level of resilience had moderate acceptability. The range of resilience varied between 0.04–0.11 with an average of 0.09. In the context of *chapatti-*making quality, resilience is an important textural attribute that influences the overall eating experience. This gives *chapattis* a pleasant and springy texture that is desirable to the consumers (47).

Correlation analysis was also performed between the characteristics of texture (hardness, springiness, cohesiveness, gumminess and resilience) and the overall acceptability. It was found that two characters, *viz.*, hardness (−0.455) and chewiness (−0.443) had a significant but negative correlation with overall acceptability (Supplementary Table S4). Boukid (48) in a review on flatbreads stated that an increase in gumminess and chewiness in these flatbreads leads to detrimental effects on overall texture resulting in a lower acceptability rate by the consumers.

### Pasting properties of maize flour

**Figure 2:**
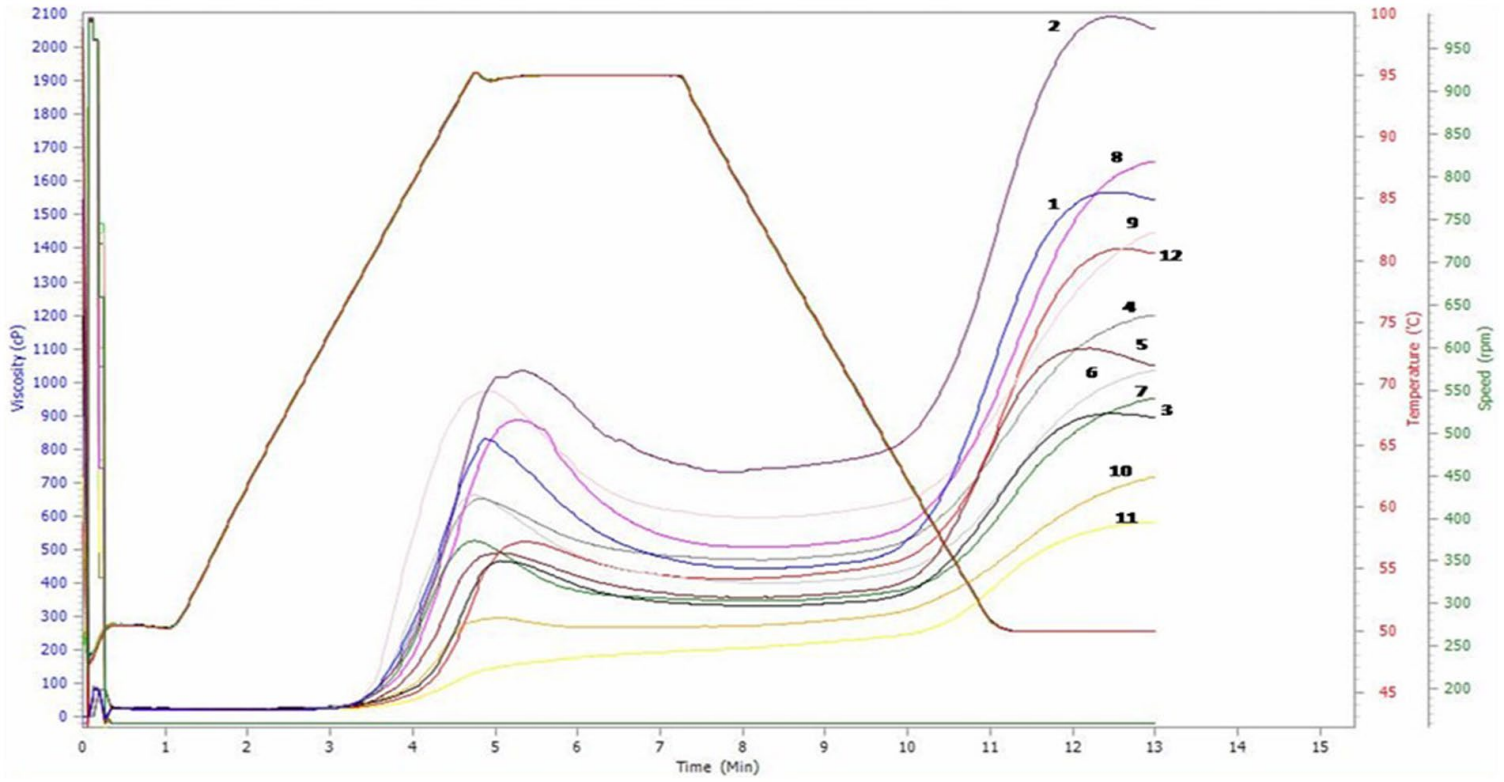
Pasting properties of maize flour [The lines of different colours in the graph represent different samples used in the study. Numbers 1 to 9 represent WHM 1 to WHM 9; 10, 11 and 12 represent yellow landrace, white landrace and check Bio 605, respectively; the X-axis represents the time taken in minutes; the Y-axis represents the range of viscosity (CP), temperature (°C) and speed (rpm), respectively].

The viscosity of the paste was assessed to examine the alterations occurring in the starch throughout the extrusion process (49). The pasting properties of the flour samples are presented in Table 7 and the graph in Figure 2.

**Table 7:** Pasting properties of white maize flour.

S. No.	Sample name	Peak viscosity (cP)	Trough viscosity (cP)	Breakdown viscosity (cP)	Final viscosity (cP)	Setback viscosity (cP)	Peak time (min)	Pasting temp (°C)
1	WHM 1	833.00 ± 5.31^d^	444.00 ± 4.00^e^	389.00 ± 5.57^a^	1547.00 ± 6.24^c^	1103.00 ± 2.65^c^	4.87 ± 0.02^de^	80.60 ± 2.56^de^
2	WHM 2	1036.00 ± 2.64^a^	731.00 ± 6.00^a^	305.00 ± 7.81^c^	2057.00 ± 3.00^a^	1326.00 ± 5.67^a^	5.33 ± 0.114^b^	82.30 ± 2.20^cde^
3	WHM 3	491.00 ± 6.65^h^	358.00 ± 2.00^h^	133.00 ± 3.00^f^	1051.00 ± 3.60^g^	693.00 ± 2.65^g^	5.07 ± 0.01^cd^	84.85 ± 4.58^ab^
4	WHM 4	653.00 ± 2.64^f^	470.00 ± 5.65^d^	183.00 ± 2.64^e^	1199.00 ± 3.00^f^	729.00 ± 3.60^f^	4.80 ± 0.20^e^	81.45 ± 2.47^de^
5	WHM 5	467.00 ± 6.92^i^	332.00 ± 2.65^j^	135.00 ± 5.00^f^	896.00 ± 2.65^j^	564.00 ± 3.60^j^	5.07 ± 0.02^cd^	88.00 ± 2.00^ab^
6	WHM 6	664.00 ± 4.35^e^	400.00 ± 5.00^g^	264.00 ± 4.00^d^	1034.00 ± 6.24^h^	634.00 ± 2.00^h^	4.73 ± 0.17^e^	80.65 ± 0.57^de^
7	WHM 7	527.00 ± 1.73^g^	347.00 ± 2.65^i^	180.00 ± 4.58^e^	951.00 ± 3.60^i^	604.00 ± 4.58^i^	4.73 ± 0.11^e^	82.30 ± 2.08^cde^
8	WHM 8	887.00 ± 3.46^c^	508.00 ± 2.00^c^	379.00 ± 2.65^b^	1657.00 ± 2.00^b^	1149.00 ± 2.65^b^	5.27 ± 0.60^bc^	84.00 ± 2.65^bcd^
9	WHM 9	974.00 ± 3.60^b^	597.00 ± 2.64^b^	377.00 ± 2.64^b^	1446.00 ± 2.00^d^	849.00 ± 5.57^e^	4.93 ± 0.13^de^	79.00 ± 1.05^e^
10	Solan-L (Yellow Landrace)	297.00 ± 1.73^j^	268.00 ± 2.65^k^	29.00 ± 2.65^h^	715.00 ± 3.60^k^	447.00 ± 2.64^k^	5.00 ± 0.75^d^	87.20 ± 1.12^ab^
11	Mali-1 (White Landrace)	195 ± 5.00^k^	180.00 ± 5.00^l^	15.00 ± 1.00^i^	582.00 ± 5.57^l^	402.00 ± 6.24^l^	7.00 ± 0.12^a^	86.00 ± 2.64^abc^
12	Bio 605	524 ± 4.00^g^	412.00 ± 5.57^f^	112.00 ± 1.00^g^	1386.00 ± 3.00^e^	974.00 ± 3.60^d^	5.33 ± 0.15^b^	88.80 ± 1.84^a^
Range		195–1,036	180–731	15.00–389.00	582–2057	402–1,326	4.73–5.33	79.00–88.80
Mean		629.00	420.58	208.41	1210.08	789.50	5.18	83.76

Values are presented as mean ± standard deviation, *n* the same column, means with different alphabets in superscript indicate significant differences (*p* ≤ 0.001), Solan-L = Yellow landrace, Mali-1 = White landrace, Bio 605 = check variety.

Pasting property depends on the rigidity of starch granules which in turn affect the granule swelling potential (50). The peak viscosity ranged from 195–1,036 cP with an average of 629. 00 cP. WHM 2 (1036.00 cP) had significantly highest peak viscosity followed by WHM 9 (974.00 cP) than both yellow (297.00 cP) and white landrace (195.00 cP). Peak viscosity represents the water binding capacity of starch which often affects the quality of the final product. The high proportion of ungelatinized starch may lead to greater peak viscosity, whereas the lower peak viscosity might be due to more degradation during processing through depolymerization and molecular entanglement (51). Kaur et al. (6) also reported a range of peak viscosity ranging between 209 and 1,097 cP, which lies close to the estimated range of peak viscosity in the present study. The trough viscosity ranged from 180–731 cP with an average of 420.58 cP. WHM 2 (731.00 cP) showed significantly highest trough viscosity followed by WHM 9 (597.000 cP) than both yellow (268.00 cP) and white landrace (180.00 cP). The breakdown viscosity ranged from 15–389 cP with an average of 208.41 cP. WHM 1 (389.00 cP) had significantly highest breakdown viscosity followed by WHM 8 (397.00 cP) than both yellow (29.00 cP) and white landrace (15.00 cP). Breakdown value is related to response of starch to shear with constant heating, causing a rupture which results in a decrease in viscosity (52). The flours of hybrids with the lowest breakdown viscosities are expected to withstand high heat treatments and shear stress, and thus would be more suitable for incorporation into products required high-temperature treatment (53). In a study by Shafie et al. (54), whole grain rice varieties were studied for pasting properties and the range breakdown viscosity of 0.33–130.67 cP was obtained. Final viscosity is the viscosity of the paste after the complete cooking and cooling process or the viscosity in real use (55). The final viscosity ranged from 582–2057 cP with an average of 1210.08 cP. WHM 2 (2057.00 cP) had significantly highest final viscosity followed by WHM 8 (1,657 cP) than both yellow (715.00 cP) and white landrace (582.00 cP). The genotypes with highest final viscosity could make sure a consistent stability of products when used as a food ingredient for thickening and stabilizing roles (53). Sandhu et al. (18) studied the pasting properties of various types of maize starches and found an average final viscosity in their study of 1785 cP the setback visciosity ranged from 402-1,326 cP. WHM 8 (1149.00 cP) had significantly highest setback viscosity followed by WHM 2 (1326.00 cP), than both yellow (447.00 cP) and white landrace (402.00 cP). The lowest setback viscosity was observed in white maize check, Mali-1 (402.0 cP). However, WHM 5 showed lowest setback viscosity (564.00 cP) out of the total nine white maize hybrids. Setback viscosity is related to starch reordering and retrogradation. The low setback viscosity indicates the lower value of retrogradation. Therefore, the *chapattis* prepared from Mali-1 and WHM 5 remained fresh for a longer period of time (6). Aidoo et al. (53) reported that genotypes with highest setback value could be utilized as an ingredient in products stored under cold temperature and, also for making pasta, whereas flour samples with relatively lower setback values could also be utilized in making low viscous foods like complementary baby foods in cassava.

Similarly, Pinto et al. (56) studied several maize landraces in the regions of Brazil and found setback viscosity in the range of 689.00–1077.50 cP. The peak time ranged from 4.73–7.00 min with an average of 5.18 min. WHM 2 (5.33 min) and WHM 8 (5.27 min) had significantly higher peak times than yellow landrace (5.00 min). However, no white maize hybrid had a higher peak time than the white landrace (7.00 min). Sangeeta and Grewal (57) also reported an average peak time of 5.78 min while studying the pasting properties of maize varieties. The pasting temperatures ranged from 79°C – 88.8°C with an average of 83.76°C. No white maize hybrid had a significantly higher pasting temperature as compared to both yellow landrace (87.20°C) and white landrace (86.00°C). The high pasting temperatures of the flours is an indication of their resistance towards swelling. The values in the present study fall within the range of 68.70–99.9°C; the pasting temperature was estimated by Uarrota et al. (58) while studying the pasting properties of Brazilian maize. The pasting properties of a food pertain to the alterations that take place in the food when subjected to heat in the presence of water. These modifications impact the texture, digestibility, and ultimate application of the food product (59). The germplasm with high values of peak viscosity, breakdown and setback is efficient for the preparation of pasta (60). There is a wide variability of pasting properties in the white maize lines selected for food technology analysis. This suggests the possibility of choosing the appropriate genotype to develop several food products. Other than *chapatti*, pasting properties are also important in many other food products. Flour with high peak viscosity and low final viscosity has been reported to be more resistant to retrogradation which gives a more desirable texture to some pastas (61) and other specialty products such as tortillas (62). Liu et al. (63) reported that most of the starch pasting, and thermal parameters were positively associated with diameter, spread ratio, and sensory scores, whereas negatively associated with hardness and thickness of biscuit prepared from wheat flour.

## Conclusion

The evaluation of white maize hybrids for several food characteristics revealed promising results. The study highlights the importance of developing high-yielding white maize hybrids to cater to the preferences of rural and tribal populations who rely on white maize as their staple food. The superior white maize hybrids exhibited higher TKW, which is associated with higher yields. The bulk density and hectoliter weight of the hybrids showed variations, suggesting variability in grain density and porosity. The nutritional analysis of the white maize hybrids revealed their potential as a nutritious food source. The *chapatti*-making qualities of the white maize hybrids were evaluated and some hybrids showed superior characteristics. WHM 1 performed best in terms of proximate compositional parameters. These findings suggest the potential of the white maize hybrids for use in *chapatti* production, which is a staple food in several parts of India. Sensory analysis concluded that three white maize hybrids, *viz.*, WHM 1, WHM 2, and WHM 8 performed significantly better than yellow landrace, local white landraces and the check hybrid. The highest-yielding white maize hybrid, WMH 1 with better nutrition status and higher acceptability can potentially replace existing low-yielding white maize landraces. Future research and breeding efforts should focus on developing white maize hybrids with enhanced organoleptic traits to meet the diverse needs and preferences of consumers.

## Data availability statement

The original contributions presented in the study are included in the article/Supplementary material, further inquiries can be directed to the corresponding authors.

## Author contributions

AA: Formal analysis, Investigation, Methodology, Writing – original draft. AD: Conceptualization, Writing – review & editing, Software, Supervision. RK: Resources, Writing – review & editing. SS: Resources, Writing – review & editing. NK: Methodology, Writing – review & editing. SD: Methodology, Writing – review & editing. YK: Writing – review & editing. DS: Resources, Writing – review & editing. SR: Conceptualization, Resources, Supervision, Writing – review & editing.
