# Supplementary material for: Development of high-yielding white maize hybrids with better chapatti-making quality compared to traditionally used local landraces
Source: Front Nutr. 2024 Feb 26;11:1330662. doi: 10.3389/fnut.2024.1330662 (PMC10947182; doi:10.3389/fnut.2024.1330662)
Supplement: Supplementary file 1 [file Table_1.DOCX]

**Supplementary Table 1: Details of samples used in the study**

| **S. No.** | **Pedigree** | **Sample Name** | **Nature of sample** | **Source** |
| --- | --- | --- | --- | --- |
| 1 | GM 95C3 × HKI 1378 | WMH 1 | White hybrid | ICAR-IIMR, Ludhiana |
| 2 | GM 216C3 × HKI 1378 | WMH 2 | White hybrid | ICAR-IIMR, Ludhiana |
| 3 | CML499 × HKI 1378 | WMH 3 | White hybrid | ICAR-IIMR, Ludhiana |
| 4 | CML 74 × HKI 1378 | WMH 4 | White hybrid | ICAR-IIMR, Ludhiana |
| 5 | GM 137C1 × HKI 1378 | WMH 5 | White hybrid | ICAR-IIMR, Ludhiana |
| 6 | GM 141C1 × HKI 1378 | WMH 6 | White hybrid | ICAR-IIMR, Ludhiana |
| 7 | GM 209C3 × HKI 1378 | WMH 7 | White hybrid | ICAR-IIMR, Ludhiana |
| 8 | CML 77 × HKI 1378 | WMH 8 | White hybrid | ICAR-IIMR, Ludhiana |
| 9 | CML 605 × HKI 1378 | WMH 9 | White hybrid | ICAR-IIMR, Ludhiana |
| 10 | Desi yellow Landrace | Solan-L | Yellow landrace | Solan, Himachal Pradesh |
| 11 | Local white Landrace | Mali-1 | White Landrace | Jammu and Kashmir |
| 12 | Check variety | Bio 605 | Yellow check hybrid | ICAR-IIMR, Ludhiana |

WMH are white maize hybrids developed at ICAR-IIMR

**Supplementary Table 2: ANOVA of selected white maize hybrids**

| **S. No.** | **Cross** | **Hybrids** | **Mean GY (kg/ha)** | **Superiority over the best check** |
| --- | --- | --- | --- | --- |
| 1 | GM 95C3 × HKI 1378 | WHM 1 | 8024.3 | 29.3% |
| 2 | GM 216C3 × HKI 1378 | WHM 2 | 7885.6 | 27.1% |
| 3 | CML 499 × HKI 1378 | WHM 3 | 7815.3 | 25.9% |
| 4 | CML 74 × HKI 1378 | WHM 4 | 7502.3 | 20.8% |
| 5 | GM137C1 × HKI 1378 | WHM 5 | 7492.9 | 20.7% |
| 6 | GM 141C1 × HKI 1378 | WHM 6 | 7010.4 | 13.0% |
| 7 | GM 209C3 × HKI 1378 | WHM 7 | 7003.5 | 12.8% |
| 8 | CML 77 × HKI 1378 | WHM 8 | 6900.7 | 11.2% |
| 9 | CML 605 × HKI 1378 | WHM 9 | 6815.7 | 9.8% |
|  | CD (5%) |  | 1282.6 |  |
|  | CV (%) |  | 17.5 |  |

CD (5%) = critical difference at 5% level of significance, CV = coefficient of variance

**Supplementary Table 3: Difference in proximate composition between flour and *chapatti***

| **Samples** | **Moisture (%)**  **(F)** | **Moisture (%)**  **(C)** | **Ash (%)**  **(F)** | **Ash (%)**  **(C)** | **Fat (%)**  **(F)** | **Fat (%)**  **(C)** |
| --- | --- | --- | --- | --- | --- | --- |
| WHM 1 | 5.87 ± 0.15^d^ | 43.33 ± 1.15^abc^ | 1.30 ± 0.01^h^ | 1.33 ± 0.11^cd^ | 4.83 ± 0.30^bc^ | 3.33 ± 0.15^d^ |
| WHM 2 | 8.73 ± 0.21^b^ | 40.73 ± 0.70^abc^ | 1.40 ± 0.03^gh^ | 1.90 ± 0.15^a^ | 4.47 ± 0.41^cde^ | 3.60 ± 0.30^cd^ |
| WHM 3 | 9.43 ± 0.15^a^ | 29.90 ± 5.20^d^ | 1.30 ± 0.20^h^ | 1.70 ± 0.20^abc^ | 4.40 ± 0.26^cde^ | 3.73 ± 0.21^bcd^ |
| WHM 4 | 7.73 ± 0.38^c^ | 40.50 ± 1.90^abc^ | 1.80 ± 0.20^bcd^ | 1.50 ± 0.15^bcd^ | 4.90 ± 0.10^bc^ | 3.43 ± 0.31^d^ |
| WHM 5 | 6.20 ± 0.20^d^ | 42.07 ± 2.70^abc^ | 1.71 ± 0.05^cde^ | 1.93 ± 0.15^a^ | 4.63 ± 0.23^bcd^ | 4.13 ± 0.11^ab^ |
| WHM 6 | 6.37 ± 0.40 ^d^ | 43.83 ± 1.60^ab^ | 1.57 ± 0.05^efg^ | 1.73 ± 0.12^ab^ | 4.17 ± 0.12^de^ | 3.93 ± 0.20^bc^ |
| WHM 7 | 6.23 ± 0.25 ^d^ | 42.20 ± 1.90^abc^ | 2.27 ± 0.15^a^ | 1.17 ± 0.15^d^ | 5.10 ± 0.26^b^ | 4.43 ± 0.11^a^ |
| WHM 8 | 6.07 ± 0.20 ^d^ | 44.47 ± 0.58^a^ | 2.00 ± 0.10^b^ | 1.33 ± 0.15^cd^ | 6.00 ± 0.20^a^ | 3.67 ± 0.23^cd^ |
| WHM 9 | 6.23 ± 0.41 ^d^ | 44.23 ± 1.46^ab^ | 1.50 ± 0.10^fgh^ | 1.43 ± 0.20^cd^ | 4.13 ± 0.25^de^ | 3.43 ± 0.30^d^ |
| Solan-L | 4.03 ± 0.45^e^ | 39.40 ± 1.55^c^ | 1.63 ± 0.05 ^def^ | 1.20 ± 0.15^d^ | 4.77 ± 0.32^bc^ | 3.37 ± 0.23^d^ |
| Mali-1 | 7.20 ± 0.56^c^ | 40.00 ± 1.27^bc^ | 1.70 ± 0.10^def^ | 1.43 ± 0.20^cd^ | 4.03 ± 0.21^e^ | 3.70 ± 0.20^cd^ |
| Bio 605 | 9.50 ± 0.44^a^ | 41.80 ± 1.30^abc^ | 1.90 ± 0.05^bc^ | 1.43 ± 0.11^bcd^ | 4.20 ± 0.26^de^ | 3.77 ± 0.15^bcd^ |
| **t value** | **24.21*** | | **1.24** | | **5.15*** | |
| **Samples** | **Protein (%)**  **(F)** | **Protein (%)**  **(C)** | **Fiber (%)**  **(F)** | **Fiber (%)**  **(C)** | **TCC (%)**  **(F)** | **TCC (%)**  **(C)** |
| WHM 1 | 9.40 ± 0.40^ab^ | 9.47 ± 0.21^b^ | 1.83 ± 0.20^ab^ | 1.40 ± 0.17^de^ | 76.77 ± 0.50^bc^ | 84.47 ± 0.14^ab^ |
| WHM 2 | 9.31 ± 0.36^b^ | 9.43 ± 0.21^b^ | 1.30 ± 0.26^bc^ | 1.53 ± 0.21^cd^ | 74.79 ± 0.53^efg^ | 83.53 ± 0.45^c^ |
| WHM 3 | 9.10 ± 0.10^bc^ | 9.33 ± 0.15^b^ | 1.90 ± 0.30^a^ | 1.93 ± .0.15^ab^ | 73.87 ± 0.64^fg^ | 83.33 ± 0.61^c^ |
| WHM 4 | 8.73 ± 0.25^cd^ | 9.53 ± 0.25^b^ | 1.70 ± 0.17^ab^ | 1.53 ± 0.06^cd^ | 75.13 ± 0.60^de^ | 84.00 ± 0.51^bc^ |
| WHM 5 | 9.87 ± 0.32^a^ | 10.15 ± 0.18^a^ | 1.10 ± 0.10^c^ | 1.07 ± 0.23^e^ | 76.50 ± 0.64^bcd^ | 82.72 ± 0.53^d^ |
| WHM 6 | 9.34 ± 0.20^b^ | 9.50 ± 0.17^b^ | 1.03 ± 0.20^c^ | 1.10 ± 0.00^e^ | 77.53 ± 0.26^ab^ | 83.73 ± 0.15^c^ |
| WHM 7 | 8.69 ± 0.25^cd^ | 8.73 ± 0.21^de^ | 2.27 ± 0.30^a^ | 1.80 ± 0.10^ab^ | 75.45 ± 0.63^cde^ | 83.87 ± 0.35^bc^ |
| WHM 8 | 9.13 ± 0.15^bc^ | 9.23 ± 0.20^bc^ | 1.77 ± 0.26^ab^ | 1.67 ± 0.11^bc^ | 75.04 ± 0.63^ef^ | 84.10 ± 0.59^bc^ |
| WHM 9 | 8.53 ± 0.35^d^ | 8.37 ± 0.31^e^ | 1.93 ± 0.15^a^ | 1.73 ± 0.11^abc^ | 77.67 ± 1.00^ab^ | 85.03 ± 0.23^a^ |
| Solan-L | 9.33 ± 0.15^b^ | 8.87 ± 0.32^cd^ | 1.97 ± 0.32^a^ | 1.93 ± 0.06^a^ | 78.27 ± 0.25^a^ | 84.63 ± 0.40^ab^ |
| Mali-1 | 9.23 ± 0.20^bc^ | 9.30 ± 0.26^bc^ | 1.90 ± 0.26^a^ | 1.53 ± 0.05^cd^ | 75.94 ± 1.00^cde^ | 84.03 ± 0.32^bc^ |
| Bio 605 | 9.03 ± 0.28^bcd^ | 9.30 ± 0.17^bc^ | 1.90 ± 0.21^a^ | 1.73 ± 0.06^abc^ | 73.47 ± 1.05^g^ | 83.77 ± 0.15^bc^ |
| **t value** | **1.49** | | **2.27*** | | **20.94*** | |

t _tab_ 2.20 at p ≤ 0.05. TCC= Total carbohydrate content, values are presented as mean ± standard deviation, in the same column, means with different alphabets in superscript indicate significant differences (p≤ 0.001), Solan-L=Yellow landrace, Mali-1= White landrace, Bio 605=check variety.

**Supplementary Table 4: Estimation of correlation analysis between overall acceptability with characters of hardness**

|  | **OA** | **Hardness** | **Springiness** | **Cohesiveness** | **Gumminess** | **Chewiness** | **Resilience** |
| --- | --- | --- | --- | --- | --- | --- | --- |
| **OA** | 1 | -0.455^*^ | -0.031^NS^ | -0.205^NS^ | -0.351^NS^ | -0.443^*^ | -0.263^NS^ |
| **Hardness** |  | 1 | 0.062^NS^ | -0.236^NS^ | 0.260^NS^ | 0.137^NS^ | 0.230^NS^ |
| **Springiness** |  |  | 1 | -0.080^NS^ | -0.078^NS^ | -0.388^NS^ | 0.234^NS^ |
| **Cohesiveness** |  |  |  | 1 | 0.191^NS^ | 0.476^*^ | 0.068^NS^ |
| **Gumminess** |  |  |  |  | 1 | 0.400^NS^ | -0.133^NS^ |
| **Chewiness** |  |  |  |  |  | 1 | 0.050^NS^ |
| **Resilience** |  |  |  |  |  |  | 1 |

OA=Overall acceptability, significant at p=0.05; NS= non-significant
