# Supplementary material for: Development of high-yielding white maize hybrids with better chapatti-making quality compared to traditionally used local landraces
Source: Front Nutr. 2024 Feb 26;11:1330662. doi: 10.3389/fnut.2024.1330662 (PMC10947182; doi:10.3389/fnut.2024.1330662)
Supplement: Supplementary file 2 [file Table_2.docx]

**Supplementary Table 2: Yield performance of selected white maize hybrids**

| **S. No.** | **Cross** | **Hybrids** | **Mean GY (kg/ha)** | **Superiority over the best check** |
| --- | --- | --- | --- | --- |
| 1 | GM 95C3 × HKI 1378 | WHM 1 | 8024.3 | 29.3% |
| 2 | GM 216C3 × HKI 1378 | WHM 2 | 7885.6 | 27.1% |
| 3 | CML 499 × HKI 1378 | WHM 3 | 7815.3 | 25.9% |
| 4 | CML 74 × HKI 1378 | WHM 4 | 7502.3 | 20.8% |
| 5 | GM137C1 × HKI 1378 | WHM 5 | 7492.9 | 20.7% |
| 6 | GM 141C1 × HKI 1378 | WHM 6 | 7010.4 | 13.0% |
| 7 | GM 209C3 × HKI 1378 | WHM 7 | 7003.5 | 12.8% |
| 8 | CML 77 × HKI 1378 | WHM 8 | 6900.7 | 11.2% |
| 9 | CML 605 × HKI 1378 | WHM 9 | 6815.7 | 9.8% |
|  | CD (5%) |  | 1282.6 |  |
|  | CV (%) |  | 17.5 |  |

CD (5%) = critical difference at 5% level of significance, CV = coefficient of variance
